# Continuity of care for children with chronic conditions: mixed methods research

**DOI:** 10.1590/1980-220X-REEUSP-2022-0232en

**Published:** 2023-01-16

**Authors:** Caroline Cechinel-Peiter, Vitória Carolini Gomes, Gabriela Marcellino de Melo Lanzoni, José Luís Guedes dos Santos, Ana Lúcia Schaefer Ferreira de Mello, Aline Lima Pestana Magalhães

**Affiliations:** 1 Universidade Federal de Santa Catarina, Florianópolis, SC, Brazil.

**Keywords:** Continuity of Patient Car, Transitional Car, Child, Chronic Diseas, Nursin, Health Management, Continuidad de la Atención al Paciente, Cuidado de Transición, Niño, Enfermedad Crónica, Enfermería, Gestión en Salud, Continuidade da Assistência ao Paciente, Cuidado Transicional, Criança, Doença Crônica, Enfermagem, Gestão em Saúde

## Abstract

**Objective:**

To analyze the continuity of care for children with chronic conditions from the transition from hospital to home.

**Method:**

Parallel-convergent mixed-methods research, with a cross-sectional study and Grounded Theory. A characterization instrument and the *Care Transitons Measure* were applied to 201 legal guardians of children with chronic conditions, and semi-structured interviews were conducted with 35 participants (among professionals and guardians). Data were combined by integration.

**Results:**

The efforts of the hospital team to promote continuity of care after discharge from the transition from hospital to home impact on the quality-of-care transition perceived by caregivers, with a mean of 89.5 (standard deviation = 12.5) points. However, the absence of formal mechanisms to guide the transition of care makes it difficult to achieve continuity of care in the health network.

**Conclusion:**

Continuity of care for children is hindered by barriers, against which hospital care professionals seek individual strategies to overcome them. It is essential to establish institutional actions and public policies aiming at the transition of care to promote continuity of care.

## RESUMO

**Objetivo:**

Analisar a continuidade do cuidado de crianças com condições crônicas a partir da transição do hospital para o domicílio.

**Método:**

Pesquisa de métodos mistos paralelo-convergente, com um estudo transversal e uma Teoria Fundamentada nos Dados. Foram aplicados um instrumento de caracterização e o *Care Transitons Measure* a 201 responsáveis legais por crianças com condições crônicas, e realizadas entrevistas semiestruturadas com 35 participantes (dentre profissionais e responsáveis). Os dados foram combinados por integração.

**Resultados:**

Os esforços da equipe hospitalar em promover a continuidade do cuidado após a alta a partir da transição do hospital para o domicílio impactam sobre a qualidade da transição do cuidado percebida pelos responsáveis, com média de 89,5 (desvio padrão = 12,5) pontos. Contudo, a ausência de mecanismos formais que orientem a transição do cuidado dificulta o alcance da continuidade do cuidado na rede de saúde.

**Conclusão:**

A continuidade do cuidado de crianças é dificultada por barreiras, frente às quais os profissionais da atenção hospitalar buscam estratégias individuais para superá-las. É fundamental o estabelecimento de ações institucionais e políticas públicas visando à transição do cuidado para promoção da continuidade do cuidado.

## RESUMEN

**Objetivo:**

Analizar la continuidad de los cuidados de los niños con enfermedades crónicas desde la transición del hospital al domicilio.

**Método:**

Investigación de métodos mixtos paralelo-convergentes, con un estudio transversal y la teoría fundamentada en datos. Se aplicó un instrumento de caracterización y la *Care Transitons Measure* a 201 tutores legales de niños con enfermedades crónicas, y se realizaron entrevistas semiestructuradas a 35 participantes (entre profesionales y tutores). Los datos se combinaron por integración.

**Resultados:**

Los esfuerzos del equipo del hospital en la promoción de la continuidad de los cuidados tras el alta de la transición del hospital al domicilio repercuten en la calidad de la transición asistencial percibida por los tutores, con una media de 89,5 (desviación estándar = 12,5) puntos. Sin embargo, la ausencia de mecanismos formales para guiar la transición de la atención dificulta la consecución de la continuidad asistencial en la red sanitaria.

**Conclusión:**

La continuidad del cuidado de los niños se ve dificultada por las barreras, frente a las cuales los profesionales de la atención hospitalaria buscan estrategias individuales para superarlas. Es fundamental el establecimiento de acciones institucionales y políticas públicas para la transición del cuidado a la promoción de la continuidad del cuidado.

## INTRODUCTION

The global rise in the prevalence of diseases and chronic health conditions in recent decades has demanded new ways of organizing health services. Considering that chronic conditions may present recurrent signs and symptoms, it is necessary to monitor the patient over time, which helps to maintain health and prevent complications and new hospitalizations^(1)^.

For comprehensive care of patients with chronic conditions to happen, it is necessary that the health services are organized in a coherent way, providing opportunities for the therapeutic trajectory to follow a continuum of care, and that these are available at an adequate time and place in the Health Care Network (HCN). Thus, continuity of care allows the patient to be fully welcomed and assisted by the timely health service^(1,2)^.

Continuity of care refers to the provision of interdependent services organized in a continuum of care over time, in which the patient is the focus of attention. It can be understood from three interrelated dimensions: relational, informational, and management continuity. These three dimensions involve the bond with the professionals involved in care, communication and information exchange over time and among the different services that assist the patient, and planning and coordination of services during the patient’s transition from one place to another. The set of these three dimensions is essential to ensure continuity of care^(3)^.

In this trajectory, the moments of transition between services constitute vulnerability for the continuity of care, requiring attention from professionals and involvement of the different management levels, especially in the transition after hospital discharge, which can result in care fragmentation^(4)^. Other elements that influence the disruption of continuity of care are the deficit of communication and integration between services in the HCN, the difficulty of access to services, the fragility in reference and counter-reference flows, and the absence of records of the services provided^(1)^. The disruption of continuity of care impacts the quality of health actions, including the increase in recurrence of hospital admissions^(5)^.

Even if necessary for the promotion of comprehensive and quality care, research on continuity of care in Brazil is still incipient, especially with regard to child health^(1)^. Similarly, in the international context, despite the specific vulnerabilities of childhood, studies on continuity of care for children still need further exploration, especially in the dimensions of informational and relational continuity^(6)^.

The prevalence of chronic conditions in childhood in Brazil is 9.5% between zero and 13 years of age^(7)^, and the mortality rate of children and adolescents with hospitalized chronic conditions is 1.3%^(8)^. Children with chronic conditions demand several shared responsibilities between family and health services. Therefore, it is noteworthy that, after a hospital stay, the child needs to have his/her demands identified and needs met, including a discharge planning that considers his/her specificities and those of the family, his/her culture, and the community where he/she lives^(9)^.

In this context, the research raised the following question: how does the continuity of care for children with chronic conditions happen after the transition from hospital to home? This study aimed to analyze the continuity of care for children with chronic conditions during the transition from hospital to home.

## METHODS

### Study Design

This is a parallel-convergent mixed-methods research^(10)^, with a cross-sectional study in the quantitative phase, and a qualitative phase that used Grounded Theory (GT), in its constructivist strand^(11)^, with equal weight assigned to the stages of the study. A mixed approach was adopted with the purpose of identifying convergences, differences or combinations between the results of the qualitative and quantitative phases, allowing a greater understanding of the problem investigated^(10)^.

### Population

Participants in the study were legal guardians of children with chronic conditions who were discharged from the hospital to their homes, and professionals involved in child care.

### Study Setting

The research was conducted from February to September 2019, in two large public hospitals in Southern Brazil (a children’s hospital and a pediatric unit of a general hospital), both of them reference hospitals for high-complexity care.

### Selection Criteria

In the quantitative stage, legal guardians older than 18 years of children with chronic conditions were included. The definition of the chronic conditions to be included in the study took into account the most prevalent chronic conditions in hospitalized children in Brazil^(8)^. Those responsible for children who had been transferred from the inpatient unit, died or were readmitted to hospital at the time of telephone contact, and those responsible who did not answer the telephone call after discharge were excluded.

In the qualitative stage, we included: legal guardians accompanying children with chronic conditions hospitalized in the institutions, and who had already experienced other hospitalizations with discharge home with the child; and health professionals involved in the care of children with chronic conditions. Parents under 18 years of age and professionals on vacation or leave of any kind were excluded.

### Sample Definition

Based on hospital admission rates, a quantitative sample was calculated, considering a confidence level of 95%, margin of error of 4 points, mean of 74.7, and standard deviation of 17.1 points^(12)^. The minimum sample size was 156 participants. Of the 395 children approached, 257 had some chronic condition. Of the legal guardians of the 257 patients, 51 did not answer the phone call, three were transferred to another unit or institution, one died, and one did not accept to participate in the second stage of data collection. Thus, 201 participants were included in this stage of the research.

In the qualitative stage, 35 participants were included, organized in three sample groups, following the theoretical sampling technique of the GT^(11)^, being ten nurses, five physicians, four nutritionists, four psychologists, three physical therapists, two social workers, one speech therapist, one hospital manager and five legal guardians. The first sample group was composed of ten nurses. The second group included 14 members of the multiprofessional hospital team (doctors, nutritionists, psychologists, physical therapists, social workers, and speech therapist). The third group was made up of 11 participants involved in home care, five of whom were responsible for children hospitalized with chronic conditions, five professionals from the outpatient setting, and one manager.

### Data Collection

Quantitative data collection was carried out in two stages. In the first, the legal guardians of children with chronic conditions were invited to participate in the research during hospitalization. If they accepted, the characterization instrument was applied and participation was requested for the second stage of data collection via telephone. In the second stage, to assess the quality of the transition of care from hospital to home, the Care Transitions Measure (CTM-15), version validated for use in Brazil, was applied, via telephone call from seven to 30 days after discharge^(13)^.

The CTM-15 is composed of 15 items on a Likert scale ranging from one to four points (strongly disagree, disagree, agree, and strongly agree). In addition, the instrument has a fifth option, Don’t Know/Not Remember/Not applicable, which is not counted. The items of the CTM-15 are divided into four factors: Preparation for self-management; Understanding about medications; Assured preferences; and Care plan. Interpretation of results is indicated by the final score, which ranges from zero to 100 points, where higher linear scale means indicate higher quality in the transition of care^(13)^.

The dependent variable was the quality of the transition of care. The categorical independent variables were: gender of the child (male; female), institution (General Hospital; Children’s Hospital), region of the municipality of residence (Same health region as hospitals; Other municipalities), and number of hospitalizations in the last 12 months (1; 2; 3; 4; 5 or more). The continuous independent variables were: age (years) and days of hospitalization (number of days).

Qualitative data collection was carried out through semi- structured interviews, conducted by the main researcher in a reserved place in the institutions. In the case of the interviews with the legal guardians, previous experiences regarding the discharge process, transition of care and continuity of care were considered, with a focus on the meanings of the daily situations that concerned the health of the child with a chronic condition.

A semi-structured script was used, with the following triggering questions: “How is your performance regarding the transition and continuity of care?”, for the professionals; and “Tell me about your routine regarding the health care of your child”, for the legal guardians. The interviews were audiorecorded and transcribed in full for later analysis, with an average duration of 24 minutes (SD = 14.7).

### Data Analysis and Treatment

The quantitative data analysis was processed through the Statistical Package for the Social Science (SPSS), version 23. The t-Student test, analysis of variance (ANOVA), and Spearman’s Correlation were applied. A confidence level of 95% (p < 0.05) was considered.

The qualitative data analysis was done through initial and focused coding. Initial coding involved coding word by word, line by line, or incident by incident, allowing for greater detail of the data. In focused coding, the most significant initial codes were contrasted and compared with each other, developing the categories and subcategories of the study. The qualitative data collection was closed when theoretical saturation was reached, when the collection of new data does not bring up new theoretical insights and the collected data support the construction of theory^(11)^.

The combination of data was done by integration, at the end of data collection from both stages, seeking to mutually strengthen the results of qualitative and quantitative research^(10)^. A meta-inference table was used, a tool that structures the joint discussion of quantitative and qualitative data and their integration^(14)^. In this article, we chose to present three categories and quantitative findings related to the continuity of care in the Health Care Network after hospital discharge, with the purpose of deepening on the most common difficulties and strategies to qualify this process.

### Ethical Aspects

The research was approved by the Ethics Committee for Research with Human Beings, opinion number 3.400.376 of 2018, in accordance with resolution 466/2012 of the National Health Council. All participants expressed their consent via signing an Informed Consent Form. The statements were identified by the letter G followed by the number corresponding to the sample group of participants, the letter E, and number indicating the order in which the interview was conducted.

## RESULTS

Most children were male (n = 110; 54.7%), residing in the same health region as the hospitals (n = 135; 67.2%), and with one hospitalization in the past 12 months (n = 127; 63.2%). Of the total patients, 90 (44.8%) were discharged from the general hospital and 111 (55.2%) from the children’s hospital. The mean age was 5.0 years (SD = 4.3), and the mean length of stay was 9.1 days (SD = 11.8).

The overall average of the CTM-15 was 89.5 (SD = 12.5) points, ranging from 74.0 to 95.6 points among the 15 items of the instrument (Table 1).

**Table 1.** Means and standard deviations according to the items of the CTM-15 (n = 201) – Florianopolis, SC, Brazil, 2019.

Items in *Care Transitions Measure*	Average	SD*
Item 1 – Agreed with the health care team on health goals and means.	88,4	14,2
Item 2 – Preferences considered when deciding health needs	87,4	9,0
Item 3 – Preferences considered when deciding Where health Needs are met	86,5	9,8
Item 4 – Had information needed for self-care	91,3	13,7
Item 5: Understands how to manage Health	92,8	15,0
Item 6: Understands warning signs and symptoms	92,5	15,2
Item 7 – Had a written plan of care	83,8	4,4
Item 8: Understands what makes the health condition better or worse	92,8	17,0
Item 9 – Understands health care responsibilities	95,5	21,9
Item 10 – Confident knowing what to do to manage care.	91,0	14,5
Item 11 – Confident could do what needed to take care of health.	90,8	15,6
Item 12: Had a written list of appointments or exams for the next weeks.	87,0	7,1
Item 13: Understand medications’ purpose.	92,7	14,0
Item 14: Understand how to take medications, including quantity and times	95,6	19,6
Item 15: Understands medications’ side effects	74,0	2,6
**Total**	**89,5**	**12,5**

*SD = Standard Deviation.

A significant difference in the quality of the transition of care was verified in the variables Gender of the patient and Region of the municipality of residence. Thus, the mean of the CTM-15 was higher for male patients (91.5 points versus 87.0 points for female patients), and for those who lived in different health regions than the hospitals (92.6 points versus 88.0 points for those who lived in cities of the same health region). No significant difference was found among the other categorical variables of the study (Table 2).

**Table 2.** Distribution of means and standard deviations of the CTM-15 according to other variables of children with chronic conditions (n = 201) – Florianópolis, SC, Brazil, 2019.

Variable		n	%	Average CTM-15	SD*	P-value
Child’s Gender	Feminine	91	45,3	87,0	15,2	**0,014** ^**†**^
	Masculine	110	54,7	91,5	9,3	
Institution	General Hospital	90	44,8	88,0	13,4	0,381^**†**^
	Children’s Hospital	111	55,2	92,6	9,6	
Region of the municipality of residence	Same region of hospitals	135	67,2	88,0	13,4	**0,007** ^**†**^
	Other municipalities	66	32,8	92,6	9,6	
Number of admissions in the last 12 months	1	127	63,2	89,9	12,1	0,888^‡^
	2	42	20,9	89,2	14,6	
	3	15	7,4	86,8	12,9	
	4	7	3,5	90,8	8,1	
	5 or +	6	3,0	91,8	7,5	
	Not informed	4	2,0	–	–	
Total CTM-15		201	100,0	89,5	12,5	

*SD = Standard Deviation ^†^t-Student Test ^‡^Analysis of Variance.

Spearman’s Correlation Coefficient between quality of transition of care and patient’s age was +0.084 (p = 0.239) and between quality of transition of care and days of hospitalization was −0.17 (p = 0.814).

In the qualitative step, difficulties and strategies in the performance of professionals who sought to promote the continuity of care from the transition from hospital to home were revealed. The categories: “Seeking to promote the continuity of care in the Health Care Network”, “Facing difficulties to promote continuity of care” and “Not acknowledging the patient’s trajectory after hospital discharge” emerged.

### Seeking to Promote the Continuity of Care in the Health Care Network

The professionals of the hospital team understand that the bond of the child with chronic conditions and the HCN will determine the maintenance of the continuity of care after discharge. In this sense, the professionals of the hospital service feel responsible for referring the patients to the necessary health actions and services, which guarantee a safe trajectory, in addition to a quality care within the limits of the hospital stay.

*Continuity of care is knowing that he will be treated here and he will have this extension of care there at the other end*. (G1E04)

*Continuity of care is that path that you started here and Primary Health Care (PHC) will provide continuity. Maybe yesterday’s problems won’t be todays, but maybe they are. So, it (PHC) will be aware of what was worked in the hospital to give continuity there in primary care.* (G1E06)

The hospital multiprofessional team is positioned as a reference of support in the care of the child in the context of the home, making itself available for guidance of the family and professionals from other points of the HCN, considering the specificities required in each case. Among the support actions mentioned by the professionals are meetings with the multiprofessional PHC team, training and availability of telephone contact, especially in cases of complex diseases.

*I was talking to the physical therapist who is there with her (patient), we were discussing her ventilation, that she needs new parameters, we discussed it. So this is cool, this bond that is being created* (G2E13)

*We make attempts, we involve primary care. It happened last month (…). Imagine something beautiful, the nurse and the technician came here, sat down, did a round with us, we made a strategy.* (G3E02)

#### Facing Difficulties to Promote the Continuity of Care

In the articulation with the other points of the HCN, it was highlighted the need for information systems and other tools that facilitate the transition of care after hospital discharge and thus promote continuity of care over time. The professionals understand the need for standardized institutional strategies, such as unified electronic medical records, protocols, information and communication systems, reference and counter-reference flows, facilitating access and avoiding loss of information.

*One thing that would be very interesting is for us to have a common medical record, from the basic unit to the hospital. They would also know what happened here, we would know information from there*. (G2E06)

*I think we could have counter-reference protocols for the health posts. Because we make these daily telephone contacts, these e-mails, but I think we could have a flow, prioritizing some diagnoses, these chronic diseases, these situations of greater vulnerability.* (G2E02)

Despite the efforts identified in the work dynamics of the hospital team, the importance of active participation of the PHC reference teams in the patient transition process was evidenced. The constant preparation and training to better care for children with chronic conditions with their specificities in PHC, with teams engaged for a safe and quality care, reflects in greater bonding and in reducing the overload of hospital services. The participants pointed out the damage of the absence of this preparation in promoting the bond of trust with the patient and family and maintaining continuity of care, while a PHC structured and engaged in care coordination contributes to communication between services, facilitating continuity of care.

*To give you an idea, the other day I was in an appointment, the doctor got up from his chair and asked me what was that thing she was wearing there. The respirator. All right, he may not have much knowledge, but it is a respirator, he should know. I was even amazed because I had to explain to him what it was* (G3E09)

*In the city where we have working PHC, it is much easier for us to get a physical therapist (…) Some municipalities have their doors open, with professionals available, and some others complicate everything.* (G3E01)

#### Not Acknowledging the Patient’s Trajectory After Hospital Discharge

Although the referrals and possible guidance are made seeking to promote the continuity of care of the child with chronic condition in the HCN, the professionals cannot be sure of the effectiveness of their conducts. Due to the lack of information about the trajectory of the child and family, the professionals show concern about their care after discharge, especially considering the need for continuous monitoring over time due to the chronic condition. At the end of the hospital stay, the hospital does not have formal strategies for monitoring the continuity of treatment. Even after discharge, these professionals feel responsible for the patient, which often generates anguish and emotional suffering.

*The most difficult thing for the chronic (patient) is to send him home after discharge, because then we often don’t have the follow-up anymore*. (G2E04)

*The patient leaves, and how will he have this care at home? Not only with the child, but also with the family. How will she be taken care of? (…) How will she manage with the medications? We worry a lot about this* (G1E02)

Upon discharge, professionals will only know about the continuity of care when faced with a readmission due to exacerbation of the chronic condition. In these episodes of exacerbation, the absence of a trusting relationship with PHC can result in the patient’s retainment in secondary and tertiary levels of care, overloading hospital services.

*We had to start having a primary care that worked well, in terms of health promotion and prevention. Even the patients don’t seek (PHC), they go straight to the hospital. They forget, leave primary care a little aside*. (G1E1)

*And I always emphasize the need for them to have contact with their municipality, with the reference center. So they don’t lose this contact (…) And then there is no need for the mother to put the child in the car and go desperately to an emergency door.* (G1E10)

**Chart 1.**
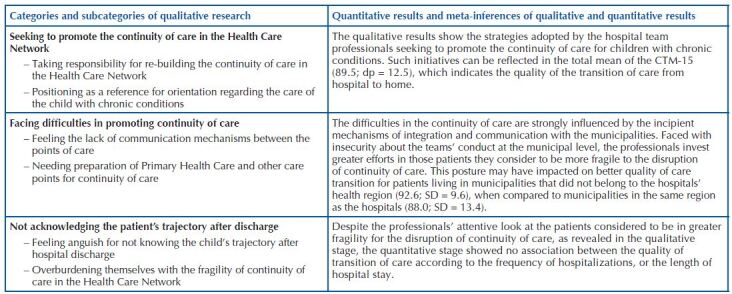
Integration of qualitative and quantitative data of the research – Florianópolis, SC, Brazil, 2019.

The integration of quantitative and qualitative data is presented in Chart 1.

## DISCUSSION

Data integration showed that the efforts of the hospital team to promote continuity of care after discharge have a positive impact on the quality of the transition of care perceived by the caregivers of children with chronic conditions. However, despite the strategies undertaken individually, the absence of formal mechanisms that guide this transition from hospital to home reflects difficulties in achieving continuity of care for children with chronic conditions in the health care network.

Continuity of care is related to the quality of health care over time, which influences a lower incidence of readmissions, greater adherence to care and patient satisfaction^(15)^. Its discussion is especially important in times of transition between services, in which continuity of care presents a great risk of interruption, resulting in fragmentation of care^(3)^.

An effective transition of care leads to a positive response to the patient, including greater adherence to care, reduction of adverse events, readmissions, and improvement in quality of life. Thus, the actions of transition of care from hospital to home have an impact on patient care in the post-discharge period, and its effectiveness impacts on good results regarding the continuity of care, with greater support during the patient’s trajectory in the HCN^(16)^, being associated with better health outcomes of children with chronic conditions^(17)^, therefore justifying the concern of professionals of the hospital team in seeking to facilitate the continuity of care over time after discharge through the transition of care.

For those reasons, the transition after hospital discharge has been a strategy for the continuity of patient care at home^(16)^. In this search, professionals try to promote the integration with PHC teams by means of different strategies, such as training of professionals and family members/caregivers, outpatient follow- up, educational strategies, discharge plan, communication of the multiprofessional team, guidance regarding the use of medications, patient monitoring by means of telephone contact or home visits, advance care planning, requisition of medications and equipment, availability of transportation, communication with other services by means of e-mail, telephone calls and printed documents^(1,18,19)^, concurring with the strategies identified in this study.

Although these are individual and not institutionalized actions, these initiatives may have influenced the quality of the transition of care perceived by those legally responsible for children with chronic conditions, which in this study showed an average of 89.5 points. The CTM-15 has not been previously used to evaluate the quality of the transition of care for children in Brazil. In the international context, the mean of the quality of care transition measured by means of the CTM-3, a shortened version of the CTM-15, was 83.7 in the United States^(20)^. Studies that used the instrument in Brazil with the adult population presented means between 69.5 and 79.0^(13,16,21)^, and in countries like the United States, Sweden and Japan, the mean of the CTM-15 ranged from 65.8 to 78.5^(22, 23, 24)^.

We can infer that the providers’ performance in the health care of children with chronic conditions includes differentiated strategies facing the understanding of the importance of continuity of care for these patients, which impacts the quality of the transition of care perceived by the patient and family.

However, these isolated strategies are not enough to ensure continuity of care over time. Among the main difficulties encountered in promoting continuity of care from the transition from hospital to home are the absence or incipiency of information and communication systems, and the divergence in the preparation and willingness of PHC in different municipal contexts to absorb the care of children with chronic conditions.

The process of articulation of the child with chronic condition with the HCN allows care over time to happen safely and easily. Several attitudes hinder the bond of the child and family with health services, including: lack of sharing of information and communication, difficulty in access to services, and poor referral and counter-referral. These gaps influence the generation of costs, the distrust of families about the resoluteness of other services of the HCN and the possible worsening of the child’s health conditions^(1)^.

The engagement of PHC professionals enables the incorporation of health promotion and disease prevention actions in a longitudinal care. Greater involvement of PHC teams influences the increase in performance, efficiency, resolution and productivity^(25)^. Otherwise, the absence of this engagement compromises the formation and maintenance of the bond of trust between the patient and family with their reference health team in the home territory.

The results of this study revealed that, in face of the different PHC settings, the hospital care professionals tend to undertake larger strategies of care transition to those patients coming from municipalities more distant from the hospitals. However, it is important to emphasize that strategies that promote access and bonding of children with chronic conditions in PHC should not come from professionals alone, configuring individual and isolated initiatives with little potential for dissemination. It is necessary, above all, investment in public policies that support these initiatives and ensure less variability of actions, with encouragement and support from different levels of management.

The findings of this study showed that the weakness of patient and family trust in PHC can lead to the overload of secondary and tertiary care services. Some of the reasons why parents seek urgency and emergency services in case of acute diseases can be due to dissatisfaction regarding the resolvability of the problems presented by the patient in PHC or by believing in a faster service in hospital care^(26)^.

The perception of users regarding the attributions of PHC is of utmost importance. It happens that many times the patient and family are unaware of the importance of being in contact with this service and have a limited view of its scope, mistakenly understanding it as a service directed to low complexity actions. On the other hand, they believe that the specialized and hospital care will meet their demands with greater amplitude, overloading these services^(27)^.

The presentation of strategies to improve the access and maintain the longitudinal link of patients with chronic conditions in PHC reflects in results in all levels of care, including the reduction of the overload of the hospital service^(28)^. Although the hospital team professionals have highlighted special concern with the continuity of care for children with chronic conditions after discharge, no difference was found between the scores of quality of transition of care from hospital to home with regards to length of stay and frequency of admissions, suggesting that, despite showing greater concern with these patients, isolated initiatives are not sufficient to reflect greater distinctions at the time of transition from hospital to home.

As limitations of the study, it is necessary to point out a possible gratitude bias on the part of the participants. Thus, the answers to the survey may have been influenced by the satisfaction of being assisted in a moment of fragility. Moreover, studies on continuity and transition of care are still preliminary, especially regarding child health care^(29)^, which limited the discussion of findings in similar contexts. However, the lack of studies that address the theme in the context of child health reinforces the novelty of this work and its contribution to the area of health and nursing. New studies are needed to deepen the understanding about the transition and continuity of care for children, supporting practices and allowing advances and improvements in these processes.

## CONCLUSIONS

The study indicated that, although the quality of the transition of care perceived by the legal guardians of children with chronic conditions was satisfactory, the gaps in communication between health services, the lack of articulation between professionals from different levels of care and the fragility of the bond between patients/guardians and PHC professionals were some elements identified as barriers to ensuring continuity of care. In this sense, unaware of the possibilities of the health trajectory of children with chronic conditions, the hospital team professionals feel responsible for these patients even after discharge, and seek, through individual initiatives, to promote the continuity of care of these patients in the PHC through transition actions.

These individual strategies may have positively impacted the quality-of-care transition perceived by the legal guardians of children with chronic conditions. However, they are not enough to guarantee the continuity of care over time in the patient’s trajectory in the SAN. The institutional formalization of mechanisms that guide the transition of care from hospital to home is necessary, reducing the variability of actions and ensuring a safe and quality transition, aiming at continuity of care.
